# Furuncular myiasis affecting the glans penis of a young boy caused by the larvae of *Cordylobia anthropophaga* (the tumbu fly): a case report

**DOI:** 10.11604/pamj.2022.42.75.35227

**Published:** 2022-05-27

**Authors:** Damilola Alexander Jesuyajolu, Paul Jesuyajolu

**Affiliations:** 1 Surgery Department, First Graceland Hospitals, Abijo, Lagos, Nigeria

**Keywords:** Myiasis, *Cordylobia anthropophaga*, glans penis, tumbu fly, case report

## Abstract

Cutaneous myiasis is endemic in West Africa, and it is most commonly caused by the larvae of Cordylobia anthropophaga. In English literature, recorded cases of this cutaneous myiasis affecting the glans penis are rare. This rarity calls for a need to consider this as a differential when looking at furuncular lesions of the glans penis. This awareness is important for practitioners who may come across this case. We report a case of furuncular myiasis of the glans penis due to the larvae of C. anthropophaga of an 11-year-old boy living in Lagos, Nigeria. The patient presented with a lesion on his glans penis, initially thought to be a boil. Upon examination, the lesion contained a single larva of C. anthropophaga. It was extracted, and the area healed well. Although endemic, furuncular myiasis of the glans penis is a very rare condition, likely related to the living circumstance of the patient. An awareness of the clinical features is important to prevent misdiagnoses of foruncular lesions that occur on the glans penis, especially in people with identified risk factors. Health education and promotion of good hygiene are important in reducing the incidence of Cordylobia anthropophaga infestation.

## Introduction

Myiasis is the infestation of living tissue by the larvae (maggots) of fly species within the arthropod order Diptera (two-winged adult flies) [1]. Furuncular myiasis is defined as the invasion of the larvae inside the healthy skin and development of a boil-like nodule or a “furuncle”. Furuncular myiasis is endemic in West Africa [2]. Myiasis is also endemic in some parts of Nigeria like the Niger Delta region [3]. The flies that produce a furuncular myiasis include *Dermatobia hominis, Cordylobia anthropophaga, Wohlfahrtia vigil*, and the *Cuterebra* species [4]. In literature, recorded cases of this cutaneous myiasis affecting the glans penis are rare. This rarity calls for a need to consider this as a differential when looking at furuncular lesions of the glans penis. This awareness is important for practitioners who may come across this case.

## Patient and observation

**Patient information:** an 11-year-old boy, accompanied by his mother, presented to the outpatient department of First Graceland Hospitals, Abijo with a six-day history of penile swelling. According to the mother, the boy had noticed it six days ago, but only just told the mother that morning. The patient complained of restlessness at night and a feeling that something was moving on his penis. No other member had this similar lesion, and it was not painful or itchy. They lived in Epe, a rural part of Lagos, Nigeria. The clothes are usually washed with water gotten from the nearby lagoon and sun-dried on a line outdoors. There was no significant past medical, surgical or family history of any ailment.

**Clinical findings:** there was no significant finding on general examination. Upon examination of the swelling around the glans penis, a small localized swelling on the left side of the glans could be seen with a whitish and mobile substance in the middle. Upon further inspection, it was clear that it was a larva of some sort. He was moved to the procedure room and petroleum jelly was applied over the slit that now contained the head of the larvae. After a few seconds, the organism began to emerge, and it was picked with a non-toothed forceps and placed on a gauze for analysis.

**Figure 1:**
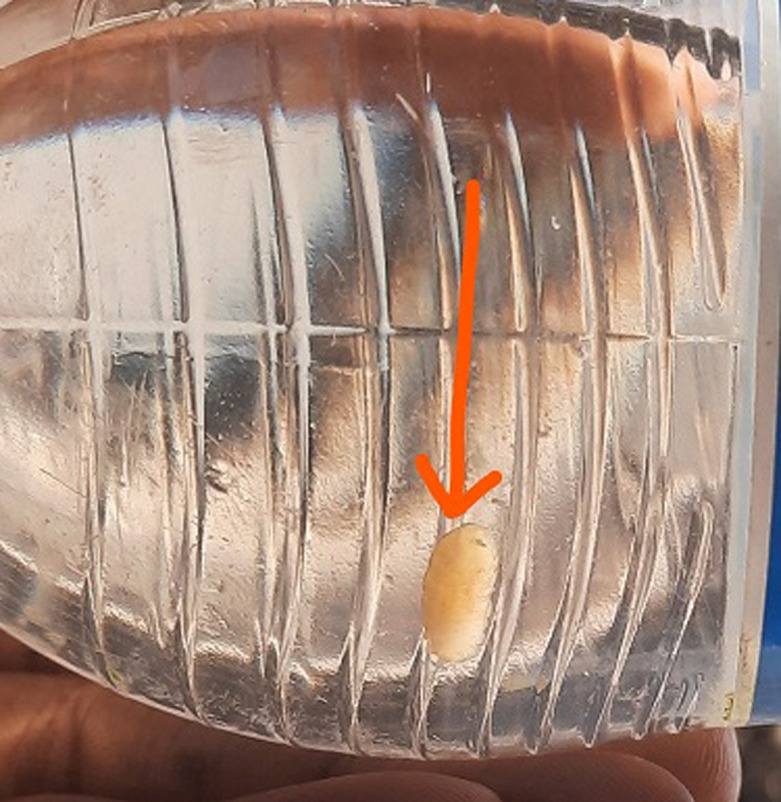
the extracted tumbu fly larvae in a plastic bottle

**Diagnostic assessment:** the microbiologist was consulted, and the specimen was examined. The characteristic morphologic features were identified (which included an incomplete peritreme and sinuous slits), it was clear that this was the larvae of the *Cordylobia anthropophaga*. The routine blood tests which included a full blood count and urea, electrolytes were normal. The organism can be seen in Figure 1.

**Diagnosis:** it became apparent that this was a case of cutaneous myiasis caused by the larvae of the *Cordylobia anthropophaga*. The patient was then inspected head to toe to make sure there was no other lesion in any other part of the body.

**Therapeutic interventions:** the patient was inspected for any similar lesion in any other part of the body. As the original lesion had been taken care of, he was prescribed oral amoxicillin 250 mg three times daily for five days for prophylaxis against a secondary bacterial infection and the patient´s mother was counselled on the diagnosis, mode of transmission and preventive measures.

**Follow-up and outcome of interventions:** the patient´s mother was told to represent in clinic with the patient in 4 weeks. The patient was lost to follow-up.

**Patient perspective:** the mother requested for the specimen to be put in a bottle for her. We took clinical pictures of the specimen, however, before she departed. She was counseled on the diagnosis and advised against spreading clothes outside where the eggs could be easily deposited.

**Informed consent:** the patient´s mother consented to the writing of this case for the purpose of enlightening the medical community.

## Discussion

The tumbu fly is the most important of the six species of *Cordylobia*, a small group of African blowflies whose maggots develop in warble-like cysts in hosts ranging from rodents to dogs and humans. The females lay eggs singly in dry sand or dirt contaminated with host urine or feces, following which the eggs hatch and eventually penetrate the host. Contact with a host allows the maggots to attach and invade the skin [5]. When the host has prolonged contact with the soil, the maggots usually have enough time to burrow into the skin of the host. They could, however, hatch on dirty and wet clothes that have fallen to the floor. In this case, the patient had a history of playing in a dirty area naked, often lying down for prolonged periods in the soil. Like in this case, when the larvae burrow into the skin, within the first 24 hours of initial contact with the host, a papule, usually pruritic, develops. It grows to form a circumscribed lesion that is often mistaken for a boil. The most posterior part of the organism can sometimes be seen through the center of the lesion. Diagnosis of furuncular myiasis is easily done based solely on clinical grounds, especially in regions where the disease is endemic [6]. Because of the resemblance to other cutaneous skin infections like furunculosis and abscesses, the presentation can be delayed, and sometimes, wrong treatment may be administered [7]. The larva, when fully mature, leaves the skin and there are usually no long-term sequelae. It is usually treated with the application of petroleum jelly to the lesion in a bid to cut off the oxygen supply to the organism. This forces them to find an oxygen source and in doing so, they move close to the central punctum of the lesion, making extraction easy. The mother was counseled as against spreading clothes outside as well as on the soil. The boy was also counseled against playing naked and health hygiene was promoted.

## Conclusion

Proper enlightenment and awareness of those at risk are important to the prevention of this condition. Because of its likeness to other skin lesions, it is important for medical professionals to be aware of this condition and to always consider it as a differential in endemic regions like Nigeria.
